# MetaNET - a web-accessible interactive platform for biological metabolic network analysis

**DOI:** 10.1186/s12918-014-0130-2

**Published:** 2014-12-05

**Authors:** Pankaj Narang, Shawez Khan, Anmol Jaywant Hemrom, Open Source Drug Discovery Consortium, Andrew Michael Lynn

**Affiliations:** School of Computational & Integrative Sciences, Jawaharlal Nehru University, New Delhi, 110067 India; The Open Source Drug Discovery (OSDD) Consortium, Council of Scientific and Industrial Research, Anusandhan Bhavan, 2 Rafi Marg, New Delhi, 110001 India

**Keywords:** Flux balance analysis, Metabolic network, Systems biology, *in silico* gene knock-out, Perturbation analysis

## Abstract

**Background:**

Metabolic reactions have been extensively studied and compiled over the last century. These have provided a theoretical base to implement models, simulations of which are used to identify drug targets and optimize metabolic throughput at a systemic level. While tools for the perturbation of metabolic networks are available, their applications are limited and restricted as they require varied dependencies and often a commercial platform for full functionality. We have developed MetaNET, an open source user-friendly platform-independent and web-accessible resource consisting of several pre-defined workflows for metabolic network analysis.

**Result:**

MetaNET is a web-accessible platform that incorporates a range of functions which can be combined to produce different simulations related to metabolic networks. These include (i) optimization of an objective function for wild type strain, gene/catalyst/reaction knock-out/knock-down analysis using flux balance analysis. (ii) flux variability analysis (iii) chemical species participation (iv) cycles and extreme paths identification and (v) choke point reaction analysis to facilitate identification of potential drug targets. The platform is built using custom scripts along with the open-source Galaxy workflow and Systems Biology Research Tool as components. Pre-defined workflows are available for common processes, and an exhaustive list of over 50 functions are provided for user defined workflows.

**Conclusion:**

MetaNET, available at http://metanet.osdd.net, provides a user-friendly rich interface allowing the analysis of genome-scale metabolic networks under various genetic and environmental conditions. The framework permits the storage of previous results, the ability to repeat analysis and share results with other users over the internet as well as run different tools simultaneously using pre-defined workflows, and user-created custom workflows.

## Background

Systems biology investigates the components of complex biological networks and can pinpoint drug targets through a combination of experimental and computational research [1]. Over the past few years, various approaches have been actively developed which attempt to provide a systems level analysis of these networks. Most of these approaches use system dynamics while others are based on a static representation of the networks which together form the core of systems biology [2]. Flux balance analysis (FBA) is a commonly used method in the field of systems biology for the quantitative simulation of metabolic networks using steady state stoichiometric models [3]. It is based on a constraint-based mathematical approach to calculate systemic phenotypes in the form of reaction fluxes. These fluxes are then used to interpret the metabolic capabilities of the system. The method’s widespread use is largely due to its independence from kinetic parameters, requiring only the stoichiometry of metabolic reactions.

Fundamentally, FBA requires four steps: (i) reconstruction of the metabolic network (ii) obtaining the stoichiometry matrix of the metabolic network (iii) defining the objective function and biochemical relevant constraints and (iv) optimization [4]. Reconstruction involves generating a network by identifying, compartmentalizing and interconnecting the components *i.e.* genes, proteins, reactions and metabolites involved in various activities of the network. The reconstructed network is then converted into a matrix S_m×n_ where *m* rows represent metabolites, *n* columns represent reactions and each element of the matrix represents the stoichiometric coefficient of the metabolite in the corresponding reaction. The dynamic mass balance of the metabolic system is

$$ \frac{dx\ }{\mathrm{dt}}=S.v $$

where ν_*n×*1_ = [v_1_, v_2_, ..... v_*n*_] is the vector of the unsolved fluxes and *×*_*n×*1_ is the vector representing metabolite concentration. At steady state, the flux distribution is defined by a system of linear equations as

$$ \frac{dx\ }{\mathrm{dt}}=S.v=0 $$

In general, for a biological metabolic system the number of reactions is more than the number of metabolites leading to an under-determined solution of steady state. Therefore, additional biologically relevant constraints are imposed on fluxes of reactions in order to find substantive solutions of the system.

$$ 0\le\ {v}_i\le \infty $$

$$ -\infty \le\ {b}_i\le \infty $$

where *ν*_i_ represents internal fluxes and b_i_ signifies the exchange fluxes in the system. Next, a reaction of interest is defined as the objective function which formulate FBA as the following linear programming problem.

$$ \mathbf{maximize}\ {\boldsymbol{C}}^{\boldsymbol{T}}\boldsymbol{v}\kern0.5em s.t.\kern0.5em \boldsymbol{S}.\boldsymbol{v} = \mathbf{0} $$

where c represents a vector with the reaction of interest set to one, while all other reactions are set to zero [5,6] . Examples of objective functions are maximization or minimization of biomass, ATP production or metabolite production. FBA has been successfully applied for predicting growth and metabolic by-product secretion in *E.coli* [7].

Over the last decade, FBA and other methods for metabolic network analysis have been implemented through various resources [8,9]. The most prominent resources include the COBRA toolbox [10], Metatool [11], FluxAnalyzer [12], MetaFluxNet [13], SNA [14] and CellNetAnalyzer [15], a successor of FluxAnalyzer. COBRA, Metatool and CellNetAnalyzer require the proprietary MATLAB environment while SNA requires MATHEMATICA for their functionality. In addition to these commercial resources, many open source applications were also developed such as YANAsquare implemented in Java [16], Linear Inverse Model [17], sybil [18] and BiGGR [19] that run within the R environment [20]. Another open source package is the Java-based System Biology Research Tool (SBRT) that has only a basic graphical user interface [21]. All of these packages are of great value for researchers, but are stand-alone applications which often require installation of external dependencies and regular updates with time. On the other hand, web-based applications are platform-independent and require only internet access to construct and analyze networks. In the context of web-based applications, FAME [22] and MicrobesFlux [23] provide facilities to create, edit and analyze genome scale model for different micro-organism obtained from the KEGG database [24]. Flux-P is another efficient web-based tool for automating and standardizing ^13^C-based metabolic flux analysis, using the Bio-jETI workflow framework [25]. Webcoli [26] provides a framework for reconstructing a genome-scale metabolic model of E.coli. MetaNetX [27] is a valuable tool which offers analysis of several metabolic networks from BiGG [28] and MetRxn [29] in its repository. Existing web-based tools contain only pre-defined applications to simulate network properties. We have introduced a web accessible platform MetaNET, which is open source, user friendly and promises to simulate complex large metabolic networks without the requirement of typical hardware or software configuration at the user’s site. In addition to being web-accessible and platform independent, MetaNET offers a workflow editor to connect various tools together, along with a data-library to enable the sharing of data-sets. The framework contains a number of primitive tools which can be used stand-alone or combined to create higher level solutions by the user. This extensibility is showcased by the development of sample workflows that cover common metabolic simulations. A comparison of basic functionality of MetaNET with other existing stand-alone and web-based applications is provided as Table 1.

**Table 1:** **Features comparison of MetaNET with similar software packages**

**Features**	**Installation requirement**	**External dependency**	**GUI**	**Programming knowledge**	**Workflow support**	**Functions**			
**Tools**						**FBA**	**FVA**	**Choke points analysis**	**Chemical species participation**
MetaNET	N	N	Y	N	Y	Y	Y	Y	Y
COBRA	Y	Y	N	N	N	Y	Y	N	N
FAME	N	N	Y	N	N	Y	Y	N	N
PathwayAnalyzer	Y	N	N	N	N	Y	N	N	N
Webcoli	N	N	Y	N	N	Y	N	N	N
CellNetAnalyzer	Y	Y	Y	N	N	Y	N	N	N
SBRT	N	N	N	Y	N	Y	Y	N	N
YANA Square	Y	N	Y	N	N	Y	N	N	N
Microbes flux	N	N	Y	N	N	Y	N	N	N
Metatool	Y	Y	N	Y	N	Y	N	N	N
MetanetX	N	N	Y	N	N	Y	Y	Y	N

## Implementation

**Figure 1:**
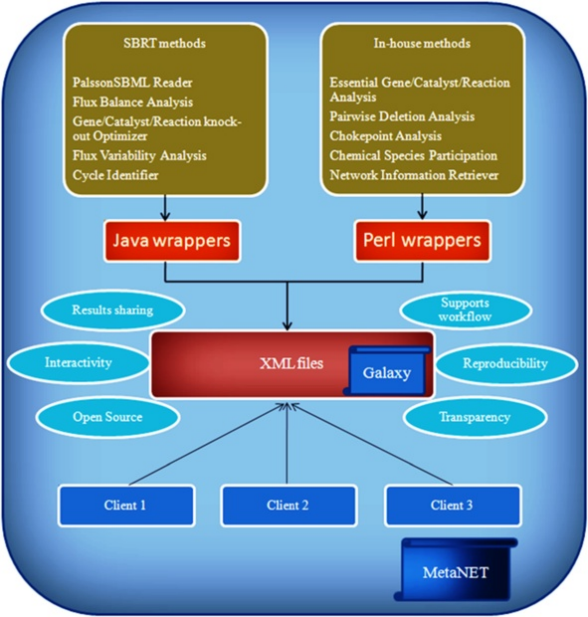
**A schematic diagram representing the different components of MetaNET.** The client–server architecture of MetaNET was developed using Galaxy framework and System Biology Research Tool (SBRT) as its main components. The functions of SBRT were called using Java wrappers and integrated with Galaxy using XML files. Additionally, the functions not present in SBRT were implemented using Perl and integrated using XML files.

MetaNET has been implemented with different functionalities of the SBRT under a GNU/Linux operating system. Although, many processes to analyze biological networks are available in SBRT, it was designed with a basic non-interactive GUI which hinders its widespread use within the research community. More importantly, users of SBRT have to create various input files manually or through programs, which are time-consuming and prone to error while handling large metabolic networks. To overcome these limitations, we extended SBRT with a user friendly platform for metabolic network studies by integrating SBRT functions with the open source platform Galaxy, available over the internet [30]. Galaxy provides a web accessible platform to integrate different command line tools to make it more interactive. Along with processes of SBRT, we have also implemented some new tools using Perl and Java languages to provide a wide range of functionalities within a single platform (Figure 1). The R Language for statistical computing is also integrated with the system, and used to provide graphical output.

### (i) Installation of tools

All the three applications: Galaxy, SBRT and R were installed on a development machine running CentOS 6.5 with the prerequisites– python version 2.6.6 & Java Development kit. Galaxy was downloaded from http://wiki.galaxyproject.org/Admin/Get%20Galaxy & installed intentionally as a normal user – “galaxy”, to permit easy migration and for reasons of security. The application runs using HTTP with a user-specified port, and does not need root privileges. This results in the easy migration by porting the entire user space to another system and as root privileges are not required, it also secures the base operating system. SBRT was downloaded from www.ieu.uzh.ch/wagner/software/SBRT and installed with the same user i.e. “galaxy”. Sybil [18] was used to find exchange reactions from SBML file. R is available as an installable package on most standard GNU/Linux operating systems, and was added by updating the package on the base operating system.

### (ii) Application development

**Figure 2:**
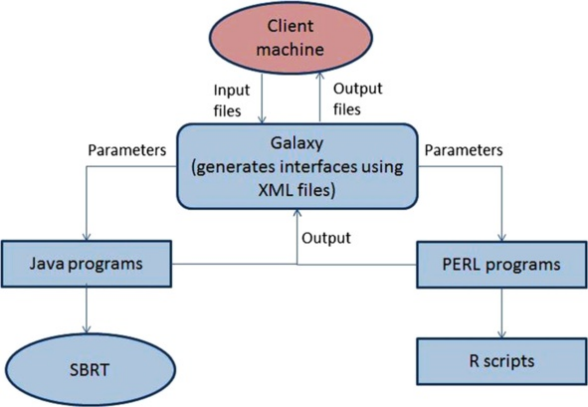
**Data flow within MetaNET.** Galaxy creates interfaces based on tool configuaration XML files for input of various parameters. User provides these parameters, input files and executes the tool through client machine. In turn, Galaxy passes these files to Java or Perl wrappers to run SBRT function or R scripts and returns results back to the client machine.

The application was implemented using XML files, Java and Perl wrappers at the following levels, as shown in Figure 2.

- XML files were written for each integrated SBRT and in-house tool which include all the information required to execute the tool. Using these files, Galaxy automatically generates the user interface for user to select input files & parameters required by the tool. On execution, Galaxy calls the respective Perl/Java wrapper and runs the tool. Table 2 contains a list of available tools and their descriptions.

**Table 2:** **Description of all tools available with MetaNET**

**Tool sub-category**	**Tool name**	**Tool description**	**Source**
Upload Data	Upload file	To upload file on MetaNET.	Galaxy
**Tool category: Format Converter**			
Import to MetaNET	BiGGSBML Reader	Converts BIGG SBML Format to reactions file.	SBRT
	PalssonSBML Reader	Converts Palsson SBML Format to reactions file.	SBRT
	Metatool Input File Reader	Converts input files of Metatool into reactions File.	SBRT
Export to MetaNET	Metatool Input File Writer	Converts reaction Files into input files of Metatool.	SBRT
**Tool category: Network Information Retriever**			
Fetch IDs	Fetch Objective function	Retrieves reaction ID of objective function from SBML file.	In-house
	Fetch Gene IDs	Retrieves list of all gene Ids from SBML file.	In-house
	Fetch Reaction IDs	Retrieves list of reaction Ids from reactions file.	SBRT
	Fetch Catalyst IDs	Retrieves list of all catalysts from SBML file.	In-house
	Fetch Chemical Species (Metabolites)	Retrieves list of chemical species Ids from reactions file.	SBRT
Fetch Reaction attributes	Fetch Flux Constraints	Retrieves flux bounds from SBML file	In-house
	Fetch Reactions-Genes Associations	Retrieves reaction-gene reactions associations from SBML file.	SBRT
	Fetch Reactions-Catalysts Associations	Retrieves reaction-catalyst associations from SBML file.	SBRT
Fetch Other Info	Generate Stochiometric Matrix	Generates stoichiometry matrix.	SBRT
	Generate Linear Equations	Generates system of linear equations.	SBRT
	Fetch Reactions	Retrieves reactions without Ids.	In-house
	Fetch Reactants	Retrieves list of all reactants from the given reactions file.	In-house
	Fetch Products	Retrieves list of all products from the given reactions file.	In-house
	Fetch Equivalent Reactions	Retrieves names of all stoichiometric equivalent reactions.	SBRT
	Fetch Flux Vectors	Retrieves flux vectors from SBML file.	SBRT
**Tool category: Flux Balance Analysis**			
Single Objective Optimizer	Single Objective Optimizer	Computes optimal value of fluxes in a stoichiometric network using multiple constraints.	SBRT
Multiple Objectives Optimizer	Multiple Objectives Optimizer	Computes optimal value of multiple objectives in a stoichiometric network using multiple constraints.	SBRT
**Tool category: Perturbation Analysis**			
Single Entity Knock-outs	Reaction knock-out Optimizer	Computes effects of deleting sets of reactions from a stoichiometric network.	SBRT
	Catalyst knock-out Optimizer	Computes effects of deleting sets of catalysts from a stoichiometric network.	SBRT
	Gene Knock-out Optimizer	Computes effects of deleting sets of genes from a stoichiometric network.	SBRT
Pairwise Entity Knock-outs	Pairwise Genes Knock-out	Computes effects of deleting pairs of genes from a chemical reaction network for multi-target drug identification.	SBRT
	Pairwise Catalysts knock-out	Computes effects of deleting pairs of catalysts from a chemical reaction network for multi-target drug identification.	SBRT
	Pairwise Reactions knock-out	Computes effects of deleting pairs of reactions from a chemical reaction network for multi-target drug identification.	SBRT
**Tool category: Essentiality Test**			
Find Essential Reactions	Essential Reactions Reporter	Finds essential reactions of a network.	In-house
Find Essential Catalysts	Essential Catalysts Reporter	Finds essential catalysts of a network.	In-house
Find Essential Genes	Essential Genes Reporter	Finds essential genes of a network.	In-house
**Tool category: Flux Variability Analysis**			
Constraint Variations	Create Constraint Variation file	Creates constraint variation file for Flux Variability Analysis.	In-house
	Flux Variability Optimizer	Computes optimal value of a single objective function for multiple set of flux constraints.	SBRT
Find Flux caps	Flux Cap Identification	Creates caps for each unbounded flux in a stoichiometric network.	SBRT
**Tool category: Other Utilities**			
File Operations	Remove beginning	Removes constraints or objectives from a file.	Galaxy
	Select First	Selects first n objectives or constraints from a file.	Galaxy
	Select Last	Selects last n constraints or objectives from a file.	Galaxy
	Add constraint	Adds constraint in a constraint file.	Galaxy
	Replace constraint	Replaces constraint of particular reaction.	In-house
	Make pairs	Makes all possible pairs of genes or catalysts or reactions IDs.	In-house
**Tool category: Flux Comparisons**			
Flux Distribution Comparison	Flux Distribution Comparison	Compares flux distributions for equality within a given tolerance.	SBRT
Flux Intervals Comparison	Flux Interval Comparison	compares intervals for equality within a given tolerance.	SBRT
**Tool category: Random Generator**			
Random Generator	Random Objectives Generator	Generates random objective functions.	SBRT
	Random Constraints Generator	Generates random constraints.	SBRT
**Tool category: Flux Distribution Plots**			
Flux Optimizer Plot	Flux Optimization Plotter	Plots result of FBA optimization.	In-house
Essentiality Plots	Reactions Essentiality Plotter	Plots result of deleting sets of reactions versus objective function.	In-house
	Genes Essentiality Plotter	Plots result of deleting sets of genes versus objective function.	In-house
	Catalysts Essentiality Plotter	Plots result of deleting sets of catalysts versus objective function.	In-house
Knock-Out Plots	Reactions knock-out Plotter	Plots the results of deleting sets of reactions or genes in a stoichiometric network.	In-house
	Genes knock-out Plotter	Plots the results of deleting sets of genes in a stoichiometric network.	In-house
	Catalysts knock-out Plotter	Plots the results of deleting sets of catalysts in a stoichiometric network.	In-house
**Tool category: Chemical Species participation**			
Grouping Chemical Species	Chemical Species Participation as Reactants	Groups chemical reactions based on the given chemical species as reactants.	SBRT
	Chemical Species Participation as Product	Groups chemical reactions based on the given chemical species as products.	SBRT
Single Species participation	Single Chemical species Participation	Finds chemical reactions containing given chemical species.	In-house
Chokepoint Analysis	Find Chokepoint Reactions	finds chokepoint reactions of biological network.	In-house
**Tool category: Network Reducer**			
Reversible Reactions Breaker	Reversible Reactions Breaker	Breaks reversible reactions into pairs of irreversible “forward” and “reverse” reactions.	SBRT
Redundant Reactions Remover	Redundant Reactions Remover	Removes redundant reactions from stoichiometric networks.	SBRT
WW Network Reducer	WW Network Reducer	Reduces size of stoichiometric networks for the purpose of identifying the cycles they contain.	SBRT
MS Network Reducer	MS Network Reducer	Reduces size of stoichiometric networks for the purpose of identifying the cycles they contain.	SBRT
**Tool category: Cycles/Paths Identifier**			
SLP Cycles	SLP Cycle Identifier	Identifies cycles in stoichiometric networks.	SBRT
Extreme Paths	Extreme Path Identifier	Identifies extreme currents in stoichiometric networks.	SBRT
- For SBRT based methods, wrappers were written in Java to call functions of SBRT. These wrappers use SBRT as an application programming interface (API) by calling its respective functions.
- For in-house tools, a second set of wrappers were written in Perl that provide missing functionality in SBRT. Additional Perl scripts were also written that take input from Galaxy and generate plots in MetaNET using R.

### (iii) Creation and sharing of custom workflows

Custom workflows were designed by combining different tools using workflow editor. The framework can filter the coupling of tools for permitting only those cases where the output from one process can be used as input of the other. Sample workflows, created to perform common metabolic simulation tasks, are listed along with a description of their functionality in Table 3.

**Table 3:** **Description of workflows implemented in MetaNET**

**MetaNET published workflows**	**Description**	**No. of steps involved**
Automated Flux Balance Analysis Workflow	Used to perform FBA of metabolic network using default objective function	6
Customized Flux Balance Analysis Workflow	Used to perform FBA of metabolic network using user-defined objective function	5
Gene Essentiality Workflow	Used to investigate lethal genes	8
Reaction Essentiality Workflow	Used to investigate lethal reactions	7
Catalyst Essentiality workflow	Used to investigate lethal catalysts	8
Pairwise Genes knock-out Workflow	Used to knock-out genes pairwise	8
Pairwise Reactions knock-out Workflow	Used to knock-out reactions pairwise	8
Pairwise Catalysts knock-out workflow	Used to knock-out catalysts pairwise	8

## Results and discussion

MetaNET currently has over 50 tools grouped into different categories (Table 2; MetaNET tool panel). Besides basic features for uploading data, format conversion and data editing, tools are available for FBA optimization, perturbation analysis and others that help to predict the cell phenotype under various genetic or environmental conditions. In addition, we have implemented a wide range of novel functionalities using in-house scripts, not available with existing web-based applications for metabolic simulations. For example, a systematic gene knock-out to identify lethal genes is a common function during analysis of biological networks, iteratively replacing the flux constraint of each reaction to zero and calculating the effective biomass. With MetaNET, users can additionally perform a pairwise knock-out of genes/catalysts/reactions for multi-target drug identification, which is otherwise a laborious task experimentally [31]. This involves switching off the reaction constraints by setting them to zero before calculating the effective biomass. The reactions, constraints and gene identifiers all extracted from the SBML file by other MetaNET tools (listed under category “Network Information Retriever”) or with the user supplied list of genes provides the input file for this function “Pairwise Entity Knock-outs” listed as a separate category on the tool panel. The results of various simulations can also be visualized graphically for better interpretation using different tools listed under category “Flux Distribution Plots”.

### Model uploading and editing

MetaNET allows users to import the model network file in different formats (SBML [32], BiGGSBML [28], PalssonSBML, Metatool Format [11], or flat file) via three methods: uploading the user’s reconstructed model from a local machine, using existing models in the MetaNET data repositories or by specifying a URL to a network file from a standard database [26], ensuring the model is available for constraint based analysis. Once the model is uploaded, tools are available under “File operations” for adding, removing or replacing flux constraints. A more detailed description for various analysis tools with required input files and their formats, is available in the user manual with MetaNET.

**Figure 3:**
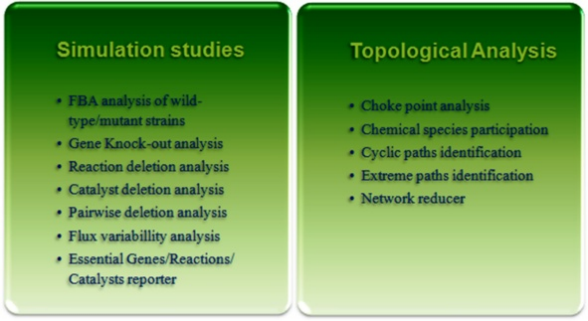
**Tool categorization within MetaNET.**

Although MetaNET can be used in various types of analysis, it can be broadly classified into two major categories: Simulation studies and Topological analysis (Figure 3).

### (i) Simulation studies

MetaNET provides various tools for simulation using FBA by applying different genetic or environmental constraints. To emphasize the core features of MetaNET, three case studies were performed with the publicly available genome-scale metabolic *in silico* model of *Escherichia coli*, (*i*JR904 GSM/GPR), from Reed *et. al*. [33]. The system consists of 761 metabolites (including external metabolites) and 1075 biochemical reactions (including transport reactions). The functionality of MetaNET has been validated in following case studies:

### Simulation of *Escherichia coli* model under aerobic conditions

**Figure 4:**
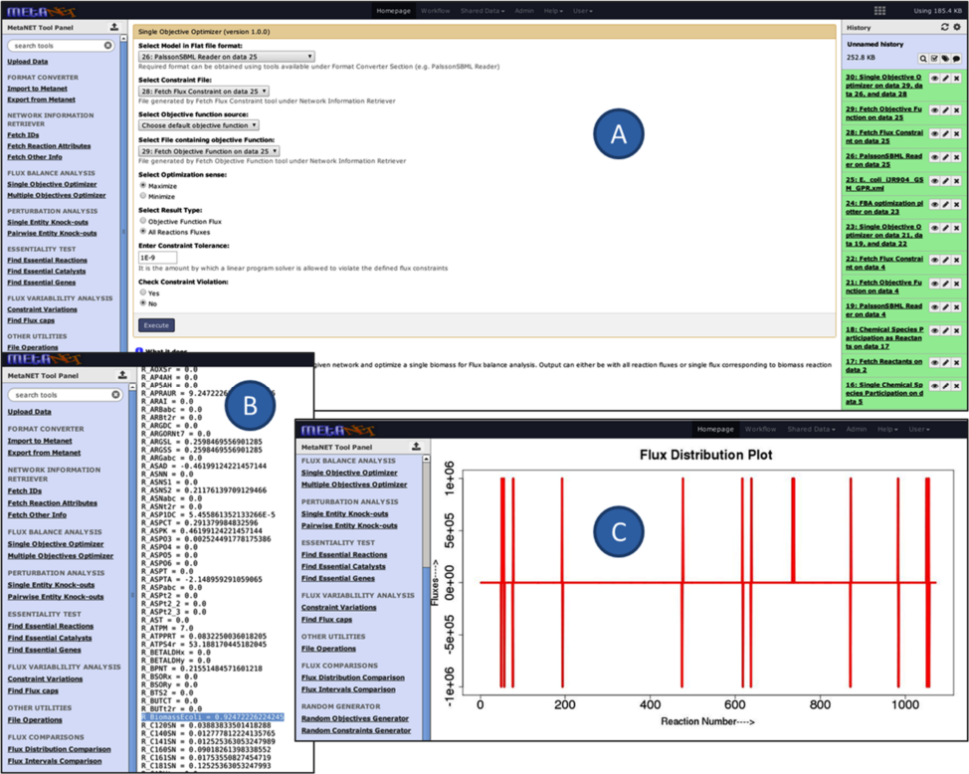
**FBA simulation of** ***in silico*** **model of** ***Escherichia coli.*** **(A)** View of “Single Objective Optimizer” for FBA of *in silico Escherichia coli* model; **(B)** Flux distribution using Biomass reaction as objective function for wildtype; **(C)** Graphical representation of flux distribution using “FBA optimization plotter”.

- To predict optimal biomass growth under aerobic conditions, the network was optimized through the biomass reaction by flux balance analysis using the “Single Objective optimizer” tool for the wild type strain (Figure 4A). The growth rate of biomass under aerobic conditions was found to be 0.92 gDW/gDW/hr while keeping glucose uptake rate fixed at 10 mmol/gDW/hr (Figure 4B). This result is in agreement with earlier published reports [34]. The flux distribution of all reactions after optimization was plotted using the “FBA optimization plotter” (Figure 4C).

### Simulation of *Escherichia coli* model under anaerobic conditions shows reduced growth

**Figure 5:**
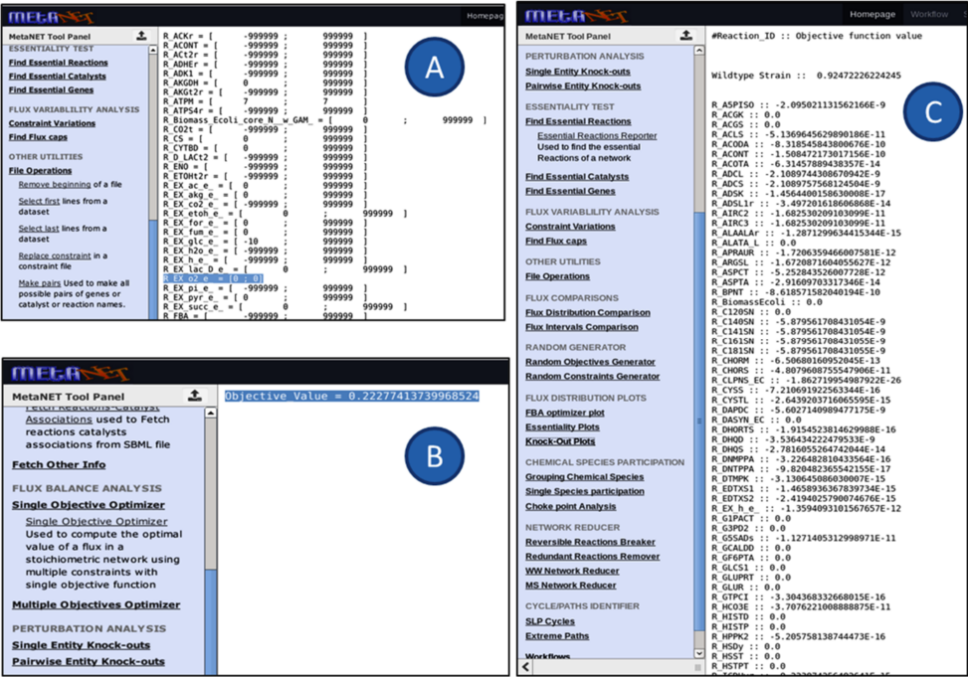
**FBA simulation of** ***in silico*** **model of** ***Escherichia coli.*** **(A)** Anaerobic conditions were created by reducing the constraint of reaction corresponding to exchange of oxygen to zero using “Replace constraint tool”; **(B)** FBA result under anaerobic conditions; **(C)** Results of reaction essentiality analysis.

- To predict optimal biomass growth under anaerobic conditions, the flux of the reaction which corresponds to exchange of oxygen from the extracellular to cytosolic compartment was constrained to zero using the “Replace constraint” tool and the network was again optimized using the “Single Objective optimizer” tool (Figure 5A). The growth rate of biomass under anaerobic conditions was found to be 0.22 gDW/gDW/hr (Figure 5B), reduction of 77% as compared to aerobic condition and similar to experimental determined values [35].

### Identification of essential reactions through perturbation analysis

- To identify the critical reactions of the network, all 1075 reactions were iteratively knocked-out and its effect on biomass was calculated using the “Reaction knock-out optimizer” tool (listed under the category “perturbation analysis”). Using “Essential Reactions Reporter”, we found 135 reactions to be essential as their deletion reduce the flux of biomass significantly (Figure 5C).

### (ii) Topological analysis

This category consists of tools designed to determine robustness and modularity based on the topology of the metabolic network. “SLP cyclic identifier” and “Extreme path identifier” are Java-based functions available within SBRT that have been have been integrated in MetaNET. Additionally, many tools are available in MetaNET which have been implemented using in-house Perl scripts. For instance, choke points are reactions present in the metabolic network which uniquely consume a particular substrate or produce a particular product [36,37]. Any enzyme which catalyzes such a reaction can be a used as drug target because blocking such enzyme results in either accumulation of particular substrate or removal of a particular product essential for survival of cell. If an enzyme catalyzes at least one choke point reaction, it can be classified as potential drug target. MetaNET provides a tool “Choke point analysis” for identification of choke point reactions in a given metabolic network for proposing drug targets. Further, MetaNET also provides a tool “Chemical species participation” to identify reactions involving metabolites of interest as reactants or products.

### Workflows support

One of the most powerful feature of MetaNET is to utilize the workflow support provided by the galaxy framework to couple tools into higher order solutions. Keeping this in mind, we designed different tools in such a way that these can be joined to create workflows in the MetaNET. Users can create custom workflows for analysis by linking appropriate tools of MetaNET using the workflow editor. The tools can only be joined if the output of one is compatible with the input of other. We have implemented eight different workflows which are generally used during metabolic network simulation (Table 3). These workflows have been publicly shared with all the users of MetaNET. Users can import these workflows to their own user-space from the “published workflows” section and extend it according to their requirements. As examples of workflows, we describe here the “Automated FBA”, “Gene Essentiality” and “Pairwise Reaction Knock-outs” workflows.

### Automated flux balance analysis workflow

**Figure 6:**
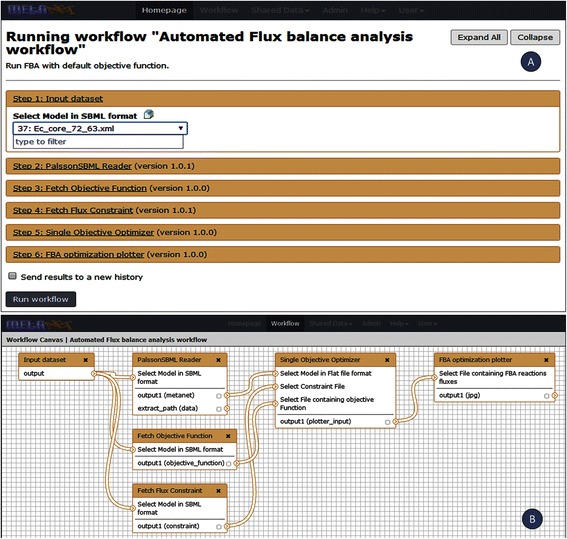
**Flux balance analysis workflow. (A)** stepwise representation **(B)** workflow representation.

This is the simplest workflow which reads the SBML file and plots the flux distribution after optimization (Figure 6). It calls the tools “PalssonSBML reader”, “Fetch flux constraints” and ”Fetch Objective function” which retrieve the reactions, corresponding constraints and objective function from the SBML file. The resulting output is passed to the “Single objective optimizer” tool which optimizes the objective function and provides the output as fluxes of all reactions. The final tool which processes the output from the earlier workflow modules is “FBA optimization plotter” that plots the flux distribution. The “PalssonSBML reader” and “Single objective optimizer” are functions of SBRT while other tools of this workflow were developed in-house.

### Gene essentiality workflow

**Figure 7:**
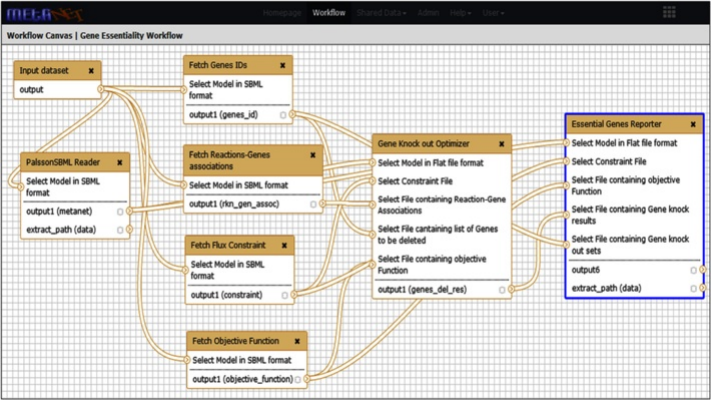
**Gene essentiality workflow.** Gene Essentiality workflow was designed by connecting various tools (shown in boxes) using workflow editor. Arrows represent the direction of flow of data from one tool to another tool.

Genes are said to be lethal if their deletion stops the growth of the organism. This workflow finds lethal genes in the given metabolic network by iteratively knocking down each gene and optimizing the metabolic network of the mutant strain (Figure 7). This workflow was designed by joining “Fetch Gene IDs”, “Fetch flux constraints”, “Fetch objective function”, “PalssonSBML Reader” and “Fetch Reaction-Gene associations” tools which parse gene identifiers, reactions constraints, objective function, reactions and gene associated with each reaction respectively from the input SBML file. The resulting output is used by the tool “Gene Knock-out optimizer” which deletes all the genes one by one and optimizes the objective function. The final tool which processes the output from the earlier workflow module is the “Essential Gene Reporter” that selects genes which reduce biomass by a user provided percentage, and is a Perl script written in-house. As an example, if a user wants a list of genes, which when knocked out, reduce the biomass of the organism by more than 90%, one can either use this shared workflow or run the tools sequentially.

### Pairwise reactions knock-outs workflow

Pairwise reactions deletion experiments require considerable time and effort to perform on a large scale. This workflow is designed to rapidly predict the biomass flux by deleting a pair of reactions simultaneously. This workflow may predict some interesting pairs of reactions whose simultaneous deletion reduce the biomass, which otherwise are found to be non-essential during single-reaction deletion analysis. It calls the tools “Fetch Reaction IDs”, “Fetch objective function”, ”“Fetch flux constraints” and “PalssonSBML Reader” which parse the reaction identifiers, objective function, reactions constraints and reactions respectively from input SBML file. As the numbers of possible pairs is very large, it first finds the choke point reactions using “Find Choke Point Reactions” tool. The resulting output i.e. reaction Identifiers of choke point reactions is used by the tool “Make Pairs” tool implemented in Perl which makes all the possible pairs of reactions. Finally, another tool “Reaction knock-out optimizer” deletes the pairs sequentially and optimizes the objective function.

## Conclusions

MetaNET is a user-friendly, extensible and platform independent framework for metabolic network analysis freely available at http://metanet.osdd.net. The framework is built with a set of tools for data management including data upload/download, format conversion, file operations and data extraction capabilities from SBML files, optimizing network using FBA, flux variability analysis [38], perturbation analysis via gene/reaction/catalyst knock-out/knock-down (single or pairwise) and visualizing results. The platform is developed by integrating the Systems Biology Research Tool with Galaxy as a proof of concept along with in-house scripts for various other functions. The rich list of tools can be interconnected through workflows to perform customized higher order simulations. End-users are encouraged to use published workflows with curated data models provided through the data libraries. Developers of data models and workflows are invited to join the mailing list set up for the purpose. The functionality of MetaNET can be further extended by including other simulation engines, and provisioning additional functions on demand from a user community, while keeping a consistent interface for models, data-sharing and functional use through shared workflows.

### Availability and requirements

**Project Name:** MetaNET

**Project home page:**http://metanet.osdd.net/

**Operating system:** Platform Independent

**Programming Language:** Language independent

**Other requirements:** Internet

**Any restrictions to use by non-academics:** None

## Abbreviations

FBA: Flux balance analysis
FVA: Flux variability analysis
SBML: System biology markup language
SBRT: System biology research tool
